# A new way to communicate science in the era of Big Data and citizen science

**DOI:** 10.1590/S1679-45082017CE4280

**Published:** 2017

**Authors:** Thiago Gonçalves dos Santos Martins, Ana Luiza Fontes de Azevedo Costa

**Affiliations:** Universidade Federal de São Paulo, São Paulo, SP, Brazil; Hospital das Clínicas, Faculdade de Medicina, Universidade de São Paulo, São Paulo, SP, Brazil

Dear editor,

In medieval times knowledge was limited to monastic libraries and access was not granted to the society. In 18th century, scientists used to travel to different countries to communicate their science in public lectures. In the same century, science become an institutionalized and professionalized activity, but it was restricted to limited communities.^(^ ^1^ ^)^ Today, although information is easily transmitted, knowledge generation still limited to universities and large laboratories that use specific communicate channels to diffuse their science. In the era of Big Data, a great amount of time is lost when scientists move to specific places to obtain knowledge and, then, diffuse it when return to their home country/city. The amount of digital data created every 2 days is an exabyte (1 billion gigabytes), the same amount of data produced by the society up to 2003.^(^ ^2^ ^)^ For this reason, many knowledge generators, the so-called “citizen scientists”, who are volunteers and do not work at large research centers have been contributing to broad knowledge by recording, for example, plants, animals, and even new stars and planets.^(^ ^3^ ^)^ “Citizen science” is breaking traditional models of science communication and favor those who were prevented to participate in science because they do not domain scientific discourse. To facilitate science communication of the current huge amount of information available, scientific journals should create a new section: collected data. The author intellectual property would be preserved by attribution of Digital Object Identifier (DOI^®^) to the published material.^(^ ^4^ ^)^ This model would improve knowledge diffusion in the era of Big Data, empower “citizen scientists” and benefit the society.
